# The Impact of Context on Affective Norms: A Case of Study With Suspense

**DOI:** 10.3389/fpsyg.2019.01988

**Published:** 2019-08-30

**Authors:** Pablo Delatorre, Alberto Salguero, Carlos León, Alan Tapscott

**Affiliations:** ^1^Department of Computer Science, University of Cadiz, Cádiz, Spain; ^2^Department of Software Engineering and Artificial Intelligence, Instituto de Tecnología del Conocimiento, Universidad Complutense de Madrid, Madrid, Spain

**Keywords:** affective norms, situational context, valence, arousal, dominance, suspense, Spanish S-ANEW

## Abstract

The emotional response to a stimulus is typically measured in three variables called valence, arousal and dominance. Based on such dimensions, Bradley and Lang (1999) published the Affective Norms for English Words (ANEW), a corpus of affective ratings for 1,034 non-contextualized words. Expanded and adapted to many languages, ANEW provides a corpus to evaluate and to predict human responses to different stimuli, and it has been used in a number of studies involving analysis of emotions. However, ANEW seems not to appropriately predict affective responses to concepts when these are contextualized in certain situational backgrounds, in which words can have different connotations from those in non-contextualized scenarios. These contextualized affective norms have not been sufficiently contrasted yet because the literature does not provide a corpus of the ANEW list in specific contexts. On this basis, this paper reports on the creation of a new corpus of affective norms for the original 1,034 ANEW words in a particular context (a fictional scene of suspense). An extensive quantitative data analysis comparing both corpora was carried out, confirming that the affective ratings are highly influenced by the context. The corpus can be downloaded as Supplementary Material.

## 1. Introduction

The cognitive-affective theory claims that human emotional response to a stimulus is mainly determined by two different information-processing systems: (a) an automatic affective system, and (b) a cognitive processing system which evaluates the information related to the stimulus (Mischel and Shoda, 1995; Moors et al., 2013). Along with visual and acoustic resources (Baumgartner et al., 2006; Dolcos and Cabeza, 2002; Eerola and Vuoskoski, 2013; Bradley and Lang, 2000), experimental studies have typically used sets of words to identify and measure what characteristics of a stimulus trigger emotional responses, and to what extent. For instance, positive/negative valence or high arousal terms seem to have a greater emotional impact than neutral terms (Maratos et al., 2000; Hamann and Mao, 2002). These terms are also more vividly remembered (Kensinger and Corkin, 2003). Other studies report differences in speed of recognition and comprehension (Estes and Verges, 2008; Citron et al., 2014b; Kuperman et al., 2014). Furthermore, the emotional response is influenced by the context in which the terms are introduced (Bokde et al., 2001; Buchanan et al., 2006; Kousta et al., 2011). This content is determinant to attribute their semantic and, hence, the subjective values of their affective features (Sperber et al., 1979; Pearson, 1998; Shaikh et al., 2007; Barrett and Kensinger, 2010).

A conventional classification for these affective features relies on three variables called valence, arousal and dominance (Russell and Mehrabian, 1977). Valence describes the degree to which a stimulus causes a positive or a negative emotion (which ranges from *pleasant* to *unpleasant*–; arousal refers the intensity or level of energy inverted in the emotion (which ranges from *calm* to *excited*–; and dominance reflects the extent of the perceived control over the emotional response when facing the stimulus (which ranges from *in control* to *out of control*) (Lang et al., 1997; Bradley, 2009; Citron et al., 2014a; Gantiva Díaz et al., 2015). Based on these affective dimensions, Bradley and Lang (1999) published the Affective Norms for English Words (ANEW), that include valence, arousal and dominance scores for 1,034 terms and their analysis. Each word was rated using a 9-point scale represented by the Self-Assessment Manikin (SAM) (Bradley and Lang, 1994), a non-verbal, pictorial assessment technique that directly measures the emotion in the three affective variables.

ANEW is the most widely referenced corpus of affective evaluations of words (Monnier and Syssau, 2014). The dataset has been replicated in other languages such as Portuguese (Soares et al., 2012), Italian (Montefinese et al., 2014), or Spanish (Redondo et al., 2007). Furthermore, different lists of words were proposed and rated in other corpora based on ANEW (Võ et al., 2006; Eilola and Havelka, 2010; Moors et al., 2013; Warriner et al., 2013; Monnier and Syssau, 2014; Schmidtke et al., 2014; Imbir, 2015, 2016; Hinojosa et al., 2016; Kapucu et al., 2018). The Affective Norms have been used to experimentally study diverse matters such as the effects of the emotional dimension of the nouns in a phrase (Fraga et al., 2012), relations between emotions and motivations (Lang, 2010), effects of depression and anxiety (Kanske and Kotz, 2012b), opinion mining (Miranda et al., 2016), sentiment and emotional categories analysis (Stevenson et al., 2007; Nielsen, 2011), adaptation to other languages through statistical regression (Wei et al., 2011), or analysis of mood in social networks (Bustamante, 2015).

Also, in a study for the detection and automatic generation of suspense framed in the development of an automatic story generator, Delatorre et al. (2017) used the ANEW dataset to determine which concepts were the best candidates to evoke suspense according to preferences of the audience. The experiment consisted of asking a number of subjects to rate the suspense provoked by a short text where different words representing decorative elements were included. Additionally, the study was repeated using an interactive 3D environment. The resulting data analysis found moderate correlations between reported suspense, and ANEW valence and dominance affective ratings. In particular, suspense increased as valence and dominance decreased and, to a lesser extent, as arousal grew.

Part of these observations are in line with the general idea of suspense found in the relevant literature (Delatorre et al., 2016b): although the existing multiple definitions of suspense largely differ in the identification and importance of its fundamental features^1^ (see Zillmann and Tannenbaum, 1980; Carroll, 1984; Ortony et al., 1990; Caplin and Leahy, 2001; Vorderer et al., 2001; Somanchi, 2003; Szilas, 2007; Abbott, 2008; Smuts, 2008, among others), there is a general agreement that suspense is triggered by the anticipation of an outcome which is mostly negative for the characters (Delatorre et al., 2018). This conceptualization can be found, for instance, in the definition by de Wied et al. (1992, p. 325), that describe(s) suspense as “an anticipatory emotion, initiated by an event which sets up anticipations about a forthcoming (harmful) outcome event for one of the main characters.” Such a common approach seems consistent with the values of the affective dimensions reported by the participants of the aforementioned experiment: decreased valence and dominance, and increased arousal.

However and although the model of Delatorre et al. (2017) revealed significant correlations in line with the mentioned concept of suspense, some other aspects seemed to challenge the validity of the experimental ratings prediction of the current affective dimensions.

First, the impact of the correlated dimensions depended on the narrative medium, that is, the correlation between the dimensions and suspense was different in the text story and in the interactive 3D environment. For the interactive 3D environment (the best case), the valence correlation reached *r* = −0.579, the arousal correlation was *r* = 0.345, and the dominance correlation was *r* = −0.498. Although this impact of valence and dominance might be considered acceptable, both failed to predict suspense evoked by concepts whose connotation varies in contexts typically associated to suspense. Thus, terms related to sanitation (such as *vomit, dirt, manure, mucus, germs*) or words as *penalty* were rated as less suspenseful than predicted. By contrast, the suspense score was higher than expected for other concepts such as *dress* or *doll*.

Secondly, the apparently low correlation of arousal in the predicted suspense was not in line with the terms often used to define suspense, such as “anticipatory arousal” (Guidry, 2005), “feeling of excitement or anxiety” (Cheong and Young, 2006), “hope and fear” (Sternberg, 1978; Ortony et al., 1990), “fearful apprehension” (Tan and Diteweb, 1996; Zillmann, 1996), or “stress” (Vorderer et al., 2001). This low correlation did not conform to previous experiments either (Brewer and Lichtenstein, 1981; Comisky and Bryant, 1982; Hoeken and van Vliet, 2000; Iwata, 2009).

As mentioned, the situational context in which the words are used influences the affective ratings (Bokde et al., 2001; Buchanan et al., 2006; Kousta et al., 2011). Since ANEW is context-independent (i.e., the words are not contextualized), the emotional response is not bound by a restricted background. This may represent a relevant difference when comparing its performance with an evaluation based on a contextualized, constraining framework. Indeed, the general affective meaning of a word is more than just a direct function of lexical affective values, since the processing of affective words is expected to interact with the surrounding context (Westbury et al., 2013; Ullrich et al., 2017). The set of cognitive and emotional reactions resulting in the absence of a context—which means the lack of effective understanding of the information environment (Bawden and Robinson, 2009)—makes it difficult to determine the relevance of the concepts (Carleton, 2012), as well as to evaluate if this relevance influences the affective assessment in the target context. This uncertainty is generated by inexactness, unreliability, and ignorance, factors which can potentially be found when no defined context is present (Funtowicz and Ravetz, 1990).

As several studies cover affective responses in non-specific contexts given words and sentence structures (Bradley and Lang, 2007; Bao et al., 2011; Imbir, 2016), other studies have experimented with generic contexts based on textual corpora (ANEW, LANG, BAWL), sound corpora (IADS) or visual corpora (IAPS) (Lang et al., 1997). The meaning of “context” varies in these studies: it can mean the emotional status of the participant (Kanske and Kotz, 2012a), tone of voice of the pronunciation of the terms (Bertels et al., 2009), visual image association (Al-Naser et al., 2015), or adjectives in Adjective-Noun-Pair structures (Gawronski et al., 2005), among others. Notwithstanding this heterogeneity, the experimental results support that, terms being presented in contexts that are either negative or imply lack of control, the affective responses are even more negative and intense than when there is no context (Bertels et al., 2009; Lambert et al., 2010; Blaut et al., 2013; Guidry, 2005; Lehne and Koelsch, 2015; Lehne, 2014). Additionally, an increase in processing speed is observed when the valence of the term is consistent with the valence of the context, which seems to confirm the effect of the congruence of both polarity and context on attention and affective evaluation (Fazio et al., 1986; Gawronski et al., 2005; Al-Naser et al., 2015; Cummings et al., 2006; Holt et al., 2009; Erk et al., 2003).

Although these results are consistent with the emotional responses to suspense also observed in Delatorre et al. (2017), none of these previously mentioned studies includes a narrative background for the terms, which would be required for a quantitative analysis of the impact of narrative contexts in story generation. Therefore, a corpus that rates affective responses to concepts in a context of suspense^2^ does not exist. As the main goal of this study, it would allow to compare the divergences with existing ANEW datasets when a specific situational background is introduced. On the other hand and particularly, it would enable the study of a model of suspense that relies on affective ratings.

On this basis, this work describes an experiment in which the three affective dimensions for the set of words included in the Spanish adaptation of the original ANEW (Redondo et al., 2007), contextualized in a suspenseful background, are rated by *N* = 206 Spanish subjects. The resulting dataset is called Spanish S-ANEW, “S” standing for “suspense.” Five different analyses of the gathered ratings are described: descriptive statistics, associations between the affective dimensions, gender differences, comparison with suspense ratings published in the aforementioned work of Delatorre et al. (2017), and relations with other psycholinguistic indices. Additionally, each analysis includes the comparison with the ANEW study performed by Redondo et al. (2007), also introducing other studies where relevant.

## 2. Methods

### 2.1. Materials and Procedure

The word set used for the experiment contained 1,034 Spanish words taken from the corpus of affective words compiled by Redondo et al. (2007). It included objective and subjective psycholinguistic indices: number of letters, number of syllables, frequency, number of orthographic neighbors, familiarity, concreteness, and imageability. Regarding the grammatical class, 713 words (68.96%) were classified as nouns, 157 words (15.18%) were adjectives, 68 words (6.58%) were verbs, and the remaining 96 words (9.28%) were included in more than one of these groups.

Participants who agreed to take part in the study received an e-mail with a spreadsheet file containing three sheets. The first sheet presented the instructions. To ensure that the participants understood them, the second sheet provided an example using the exact same set of sample words proposed by Redondo et al. (2007). Finally, the third sheet listed the 1,034 words. This sheet was divided in a first column presenting the list of the words, and three groups of columns to score valence, arousal and dominance, with the respective Self-Assessment Manikin pictogram (Bradley and Lang, 1994) in the header. Each of these three groups was composed of nine columns, visually aligned with the positions of its respective SAM figure header. Except for the headers, all the rows kept a size of 20 pixels. All the columns to score had 30 pixels, and the dimensions were separated by 40 pixels. The font used was Times New Roman, size 12 pt. The first column and row had a locked layout to ensure that subjects had the references of both the words and the SAM pictograms at all times.

Each participant was instructed to rate every word in the entire set in the three affective dimensions by placing an X in the corresponding columns, only one for each word-dimension pairing. The list of words for every file was randomly ordered, resulting in a different permutation for every participant. The evaluation had to be carried out by contextualizing each word of the set within a scene of suspense. In order to avoid limiting this context to a specific scene, scene details were omitted. Actually, the scene could be freely altered for each word at the discretion of the subject. The objective was to give freedom of choice to imagine the scene (Delatorre et al., 2017). However, and to clarify the concept of “scene of suspense,” it was described as a situation in which a victim is under a forthcoming harmful outcome event due to a threat. This description is based on de Wied et al. (1992) definition of suspense.

Additionally, the instructions illustratively described the SAM model and the three affective variables (valence, arousal, and dominance). It was also remarked that there was no right or wrong answer, although the importance of using the entire range of ratings was emphasized as well as to rate the words according to the first impression, not spending too much time with any word. Also, participants did not receive instructions regarding ambiguous words. Participants were asked to fill in and send the completed file in within 2 weeks. These instructions were similar to those provided in related studies (Bradley and Lang, 1999; Eilola and Havelka, 2010; Redondo et al., 2007; Moors et al., 2013; Montefinese et al., 2014), and are available as Supplementary Material to this article.

The study was carried out in accordance with the recommendations of national and international ethics guidelines, *Código Deontológico del Psicólogo* and American Psychological Association. The study did not present any invasive procedure, and it did not carry any risk to the participants' mental or physical health, thus not requiring ethics approval according to the Spanish law BOE 14/2007 and the ethical guidelines of authors' institutions. All subjects participated voluntarily and gave written informed consent in accordance with the Declaration of Helsinki. They were free to leave the experiment at any time.

### 2.2. Participants

An initial set of *N*_0_ = 318 undergraduate students from different fields in three public Spanish universities^3^ participated in this study. However, after the time period for sending back the questionnaires expired, 97 voluntaries did not submit a completely filled-in answer (86), or they did it after the deadline (11). Additionally, 15 more questionnaires were excluded because they had less the half of the questions answered. The rest of questionnaires were sent in time, and all of them presented a percent between 98% and 100% of questions answered. Therefore, the final set of voluntaries was composed of a total of *N* = 206 undergraduate students (123 males (59.71%), and 83 females (40.29%)). Subjects ranged in age from 18 to 37 years old (*M* = 21.26, *SD* = 2.24). All of them were native Spanish speakers.

### 2.3. Description of the Database

The resulting database is available as Supplementary Material to this article. For ease of comparison, it was organized in the same structure as published by Redondo et al. (2007), as following:

- **Number**: A numeric identifier for each of the 1,034 words, matching the original number in the ANEW dataset (Bradley and Lang, 1999).
- **E-Word**: The noun of the original English word in the ANEW dataset.
- **S-Word**: The Spanish translation of the corresponding *E-Word*.
- **Affective assessments**: For valence (SVal), arousal (SAro) and dominance (SDom), the mean values (M), and the standard deviations (SD) of suspense ratings. Data of the global sample (All) appears first, followed by data corresponding to females (Fem) and males (Mal).

## 3. Results and Interpretation

Once the data was gathered and ordered, a split-half test was conducted in order to check its reliability and consistency. The three indexes (valence, arousal, and dominance) were calculated on 1,000 different randomizations of the participants. The mean correlations between groups were high for all affective dimensions: 0.93 for valence, 0.87 for arousal, and 0.85 for dominance. This finding agrees with the previous studies of ANEW (Redondo et al., 2007; Eilola and Havelka, 2010; Moors et al., 2013; Monnier and Syssau, 2014).

Hereunder, section 3.1 details the descriptive statistics of the analysis, carried out similarly to the related literature (Bradley and Lang, 1999; Redondo et al., 2007; Soares et al., 2012; Moors et al., 2013; Warriner et al., 2013; Monnier and Syssau, 2014; Montefinese et al., 2014). Additionally, a brief study of the words that were rated the highest and lowest for each affective dimension is included. The goal of this analysis was to study numeric and semantic differences within a non-contextualized affective evaluation. The resulting deviations and their potential interpretations are presented.

Section 3.2 presents the study of associations between the affective dimensions for Spanish S-ANEW and in comparison to Redondo et al. (2007) ANEW. The results offer an explanation for the high correlation found in the Spanish S-ANEW scores against the suspense ratings published in Delatorre et al. (2017). This correlation is analyzed and described in section 3.4. Finally, gender differences and relations with other psycholinguistic indices are also analyzed, in line with the previous ANEW studies.

### 3.1. Descriptive Statistics

**Figure 1:**
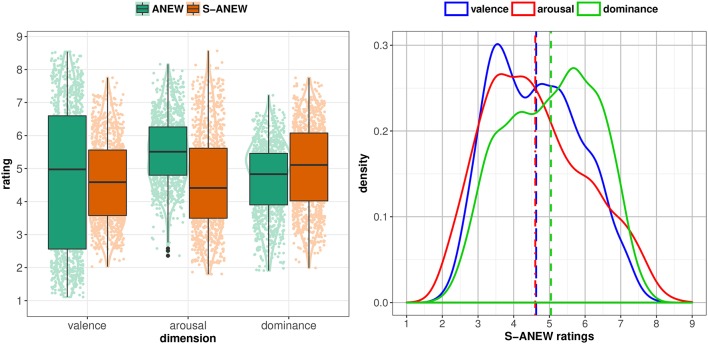
**(Left)** Box plot representing valence, arousal and dominance ratings, classified by dataset. **(Right)** Spanish S-ANEW affective dimensions density of ratings. Plot bandwidth adjust set to 1.

Table 1 shows the central tendency and variability of both Spanish S-ANEW and Redondo et al. (2007) ANEW, which are visually displayed in Figure 1 (left).

**Table 1:** Comparison of descriptive statistics between Spanish S-ANEW and Redondo et al. (2007) ANEW.

	**S-ANEW**			**ANEW**		
**Descriptive**	**val**	**aro**	**dom**	**val**	**aro**	**dom**
Mean	4.63	4.60	5.04	4.74	5.53	4.67
Stdev	1.22	1.42	1.26	2.14	1.00	1.06
Min	2.03	1.80	1.99	1.11	2.36	1.91
Max	7.75	8.56	7.75	8.54	8.16	7.22
1st Qu	3.58	3.50	4.02	2.56	4.80	3.90
3rd Qu	5.56	5.62	6.08	6.60	6.26	5.46
IQR	1.98	2.12	2.06	4.03	1.46	1.56
Kurtosis	−0.87	−0.64	−0.97	−1.36	−0.45	−0.59
Skewness	0.24	0.39	−0.12	−0.05	0.05	−0.35

The initial analysis of the central tendency revealed that ratings for valence and arousal were lower in Spanish S-ANEW than in Redondo et al. (2007) ANEW. However, the mean valence difference^4^ was small (*dif* = 0.11, *t* = 2.67, *p* <0.01), although the difference in terms of dispersion was high (2.05). Moreover, significant mean differences were found for arousal (*dif* = 0.92, *t* = 24.18, *p* <0.0001), where Spanish S-ANEW rated almost one point out of nine less than Redondo et al. (2007) ANEW in the SAM scale. By contrast, dominance ratings were just barely higher in Spanish S-ANEW than in Redondo et al. (2007) ANEW (*dif* = −0.37, *t* = −13.13, *p* <0.0001).

Figure 1 (right) shows the Spanish S-ANEW distributions of valence, arousal and dominance affective ratings. It illustrates that the distributions moderately skewed left for the valence (*Sk* = 0.24) and arousal (*Sk* = 0.39) indexes, while dominance is barely skewed right (*Sk* = −0.12). Both valence and arousal ratings trended to land in a lesser range than the mean of the words, respectively, 51.16% and 54.06%; while most of dominance ratings scored higher, in a value range greater than the mean (51.64%). As it can be seen in the figure, the respective maximum density was reached when valence got a value of 3.54 (30.13% of ratings), and 3.64 for arousal (26.63% of ratings), in both cases the deviation was higher than one point. This difference is lesser in dominance, which results in the maximum density at 5.68 (27.34% of ratings). Furthermore, the three dimensions presented a platykurtic distribution (*K*_*val*_ = −0.87, *K*_*aro*_ = −0.64, *K*_*dom*_ = −0.97), having a moderate to low concentration of values around the average and, consequently, a moderate to high scoring range.

**Figure 2:**
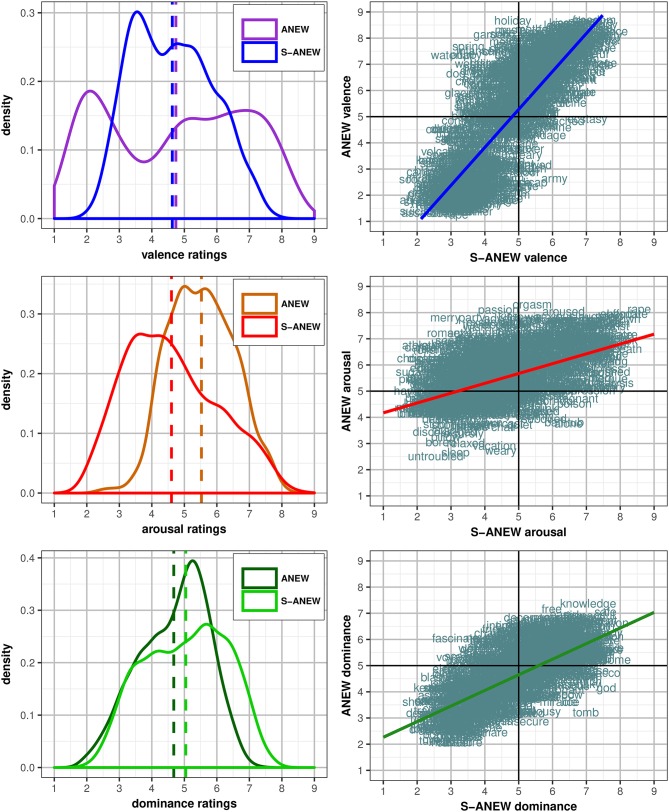
ANEW and Spanish S-ANEW comparison of valence **(Up)** arousal **(Center)**, and dominance **(Bottom)** ratings (plot density bandwidth adjust set to 1).

Additionally, the distribution of the Spanish S-ANEW ratings was individually compared with the Redondo et al. (2007) ANEW scores in each of the affective dimensions. Specifically and regarding the valence, both Spanish S-ANEW and Redondo et al. (2007) ANEW curves presented a similar shape, as shown in Figure 2 (up). However, while the distribution for the valence in Redondo et al. (2007) ANEW covered almost the entire rating scale ([1.11, 8.54]), the range of the Spanish S-ANEW had a more limited reach ([2.03, 7.75]). Therefore, the density of the rates was increased in the center of the curve. Furthermore, the Redondo et al. (2007) ANEW valence curve presented a bimodal distribution, with a higher concentration from 1.5 to 2.5, and from 5.5 to 7.5 mean ratings. Also, the peaks of the Spanish S-ANEW valence curves were located between 3 and 4, and between 4.5 and 5.5. These results revealed similarities between both distributions, where Spanish S-ANEW valence ratings seemed to be distributed in a similar way to Redondo et al. (2007) ANEW valence ratings did, but in a more confined range. As Figure 2 (up, right) shows, there is a linear uphill strong correlation (*R* = 0.826, *p* < 0.0001), and the aforementioned small mean difference (*dif* = 0.11, *t* = 2.67, *p* < 0.01) supports this observation.

By contrast, as presented in Figure 2 (center), the range of Spanish S-ANEW arousal ratings ([1.80, 8.56]) was higher than in Redondo et al. (2007) ANEW ([2.36, 8.36]), whose arousal ratings were concentrated mostly in the upper part of the scale, with prevalence around the mean (*K* = −0.45, *Sk* = 0.05). This time, the best adjusted correlation between both measures is moderate (*r* = 0.532, *p* < 0.0001).

Also, as presented in Figure 2 (bottom), the dominance dimension curves presented similarities across both datasets. Both were platykurtic distributions that concentrated the ratings around the mean, barely skewed right, and with a moderate to strong correlation (*R* = 0.709, *p* < 0.0001). However, Redondo et al. (2007) ANEW dominance ratings showed a tendency for lower affective score in the case of higher rated words, which is reflected in the resulting significant difference of means (*dif* = −0.37, *t* = −13.13, *p* < 0.0001).

In order to analyze semantic differences in the highest and lowest Spanish S-ANEW and Redondo et al. (2007) ANEW scores, two samples of the thirty highest rated terms were gathered from both datasets, respectively. Their valence scores were used to classify the terms as negative -from 1 to 4-, neutral -from 4 to 6-, or positive -from 6 to 9-, in accordance with the criteria as used by authors as Ferré et al. (2012), Monnier and Syssau (2014), and Hinojosa et al. (2016). Table 2 shows this classification, including number of words, mean, standard deviations and range, for valence, arousal and dominance. The data evidences the decreasing number of positive words in Spanish S-ANEW, most of which are in the neutral valence value range.

**Table 2:** Words classified as negative, neutral, and positive by ranges of valence, both in Spanish S-ANEW and Redondo et al. (2007) ANEW.

			**Mean (SD) and range**		
**Word type**	**Dataset**	**Words (%)**	**val**	**aro**	**dom**
Negative	S-ANEW	379 (36.65%)	3.34 (0.41)	5.68 (1.30)	4.03 (0.98)
			[2.03, 3.99]	[2.18, 8.56]	[1.99, 7.40]
	ANEW	399 (38.59%)	2.36 (0.68)	5.90 (0.94)	3.66 (0.72)
			[1.11, 3.99]	[2.58, 7.98]	[1.91, 5.50]
Neutral	S-ANEW	485 (46.91%)	4.96 (0.55)	3.96 (1.10)	5.44 (0.96)
			[4.00, 6.00]	[1.80, 7.57]	[2.94, 7.59]
	ANEW	277 (26.79%)	5.08 (0.54)	4.88 (0.74)	4.98 (0.54)
			[4.00, 6.00]	[3.23, 7.59]	[2.94, 6.49]
Positive	S-ANEW	170 (16.44%)	6.54 (0.39)	4.03 (0.97)	5.86 (0.94)
			[6.01, 7.75]	[1.86, 7.11]	[3.15, 7.75]
	ANEW	358 (34.62%)	7.13 (0.65)	5.61 (0.99)	5.57 (0.63)
			[6.01, 8.54]	[2.36, 8.16]	[3.46, 7.22]

Table 3 shows the thirty words with the lowest and highest valence dimension ratings for each dataset, around 2.5% at each end. All the lowest rated terms were mostly related to concepts that generate danger, negative emotional states, sickness, or pain, although the Redondo et al. (2007) ANEW dataset included more potential large-scale tragedies, either causes or effects as *war, accident, massacre, bomb, misery, destruction, terrorist*. Beyond that, no other semantic groups differentiated Spanish S-ANEW from Redondo et al. (2007) ANEW. Four words (13.33%) were shared between them. All selected terms in each dataset were considered within the range of negative words in the other dataset.

**Table 3:** The thirty lowest (left) and highest (right) valence rated words.

**Lowest valence**				**highest valence**			
**S-ANEW**	**val**	**ANEW**	**val**	**S-ANEW**	**val**	**ANEW**	**val**
***Suicide***^−^	2.03	Rape^−^	1.11	***Peace***^+^	7.75	***Freedom***^+^	8.54
Syphilis^−^	2.17	Dead^−^	1.17	Trust^+^	7.64	Holiday^•^	8.52
Scorching^−^	2.21	***Assassin***^−^	1.18	Liberty^+^	7.59	***Love***^+^	8.50
***Assassin***^−^	2.21	Killer^−^	1.23	***Happy***^+^	7.46	***Pleasure***^+^	8.48
Abduction^−^	2.24	Cancer^−^	1.23	Victory^+^	7.29	Kiss^+^	8.43
Invader^−^	2.25	War^−^	1.23	Truth^+^	7.28	Merry^+^	8.41
Cannon^−^	2.26	Death^−^	1.23	Miracle^+^	7.24	Friend^+^	8.41
Bees^−^	2.42	***Suicide***^−^	1.24	***Freedom***^+^	7.23	Cuddle^+^	8.41
***Funeral***^−^	2.50	Torture^−^	1.24	Angel^+^	7.22	***Happy***^+^	8.37
Spider^−^	2.56	Accident^−^	1.32	Sun^+^	7.22	Laughter^+^	8.34
Chaos^−^	2.57	Drown^−^	1.32	Adorable^+^	7.21	Valentine^+^	8.33
Afraid^−^	2.57	Massacre^−^	1.32	***Hug***^+^	7.19	Fun^+^	8.32
Betray^−^	2.57	Paralysis^−^	1.33	Cozy^+^	7.19	Optimism^+^	8.31
Shotgun^−^	2.58	Tumor^−^	1.34	Heal^+^	7.19	Free^+^	8.28
Headache^−^	2.59	Burial^−^	1.36	Useful^+^	7.18	Caress^+^	8.27
Sour^−^	2.61	Mutilate^−^	1.36	Grateful^+^	7.17	Party^+^	8.26
Jealousy^−^	2.61	Abuse^−^	1.39	Sunrise^+^	7.16	***Paradise***^+^	8.24
Foul^−^	2.61	Corpse^−^	1.41	***Pleasure***^+^	7.13	***Kindness***^+^	8.22
Arrogant^−^	2.61	***Bomb***^−^	1.42	Lucky^+^	7.13	Mother^•^	8.19
Crash^−^	2.61	Leprosy^−^	1.42	***Paradise***^+^	7.12	Family^+^	8.18
Punishment^−^	2.62	Bloody^−^	1.42	Win^+^	7.11	Music^•^	8.16
Ulcer^−^	2.63	Unhappy^−^	1.43	Rainbow^+^	7.10	Friendly^+^	8.14
Blackmail^−^	2.63	Misery^−^	1.47	Knowledge^+^	7.10	Sunlight^+^	8.14
Useless^−^	2.64	Suffocate^−^	1.48	Advantage^+^	7.10	***Hug***^+^	8.13
***Bomb***^−^	2.65	***Funeral***^−^	1.48	***Love***^+^	7.07	Hope^+^	8.11
Decompose^−^	2.65	Terrible^−^	1.49	Ecstasy^•^	7.03	***peace***^+^	8.11
Shark^−^	2.66	Depressed^−^	1.50	Triumph^+^	7.02	***Affection***^+^	8.10
Criminal^−^	2.67	Destruction^−^	1.50	***Affection***^+^	7.00	Enjoyment^•^	8.09
Coffin^−^	2.67	Sickness^−^	1.51	Humble^+^	7.00	Beach^•^	8.07
Crime^−^	2.67	Terrorist^−^	1.51	***Kindness***^+^	7.00	Life^•^	8.07

Existing words in both dataset are remarked.

+ , positive;

•, neutral; or

−, *negative word in the other dataset*.

Regarding the highest rated terms, nine words (30.00%) were shared between both datasets. Redondo et al. (2007) ANEW contained more terms related to interpersonal relations (30.00%) (*love, kiss, friend, party, mother, family, valentine, hug*), while in the Spanish S-ANEW set there were more words related to positive outcomes (20.00%) (*peace, liberty, victory, freedom, win, triumph*), and the means to achieve them (16.67%) (*miracle, useful, lucky, knowledge, advantage*).

In order to include the relation of the other dimensions with the valence, the study of the highest and lowest ratings of arousal and dominance sets of words is described in the next section.

#### 3.1.1. Interpretation of Results

The results of the demographic analysis show that the curves in both Spanish S-ANEW and Redondo et al. (2007) ANEW have similar shapes and scores, and moderate to strong correlations were found between both datasets in the three affective dimensions. The distributions of overall ratings are centered over the middle of the scale with a slight bias. The ratings also present significant differences; specifically: (a) the number of words considered positive in a context of suspense is around half the amount of the words obtained when no context is introduced, and mean values are slightly less pleasant; (b) in suspenseful contexts, subjects tend to rate pleasant terms as less pleasant, and tend to use a lower of valence (one point over nine of deviation at each end), implying a lower concept polarization according to their semantics; (c) concepts evoke less arousal in the subjects when the words are introduced in a scene of suspense, resulting a higher tendency to emotional neutrality than in the non-contextualized ratings; and (d) words that evoke the highest control tend to be rated as evoking slightly more dominance when the suspenseful context is introduced.

Therefore and in terms of valence, in a context of suspense subjects do not rate concepts as either extremely pleasant or extremely unpleasant. This contrasts with the results in experiments without context, in which subjects use a wider range in ratings. On the other hand, arousal ratings in the context of suspense present a clear displacement toward a lower segment of the rating range, slightly more expanded, and resulting in a lower density for high rating values. Finally, words with a medium-high dominance score have a higher rating for this dimension in Spanish S-ANEW than in Redondo et al. (2007) ANEW. This suggests that concepts evoking an either neutral or positive sense of control do it even more in a context of suspense.

Furthermore, an analysis of the most and the least pleasant words shows semantic differences between the contextualized and non-contextualized word sets. In a suspenseful scene, several of the most pleasant words involve positive outcomes and the means to achieve them. With suspense defined as a situation in which a victim is under a forthcoming harmful outcome event due to a threat, it is assumable that concepts related to escaping the threat are the most desirable (e.g., *peace, liberty, victory, freedom*). This trend is not present when the context is not specified, with the top-scoring pleasant words involving interpersonal relations. By contrast, in a context-less scenario, the lowest rated words are related to potential large-scale tragedies. This trend is not observed in a suspenseful context.

A plausible reason for these divergences is that the specific background allows to contextualize and to manage the different concepts in a more accurate way, reducing the uncertainty. In addition, an analysis of the elements seems to show a tendency to assign a greater arousal to concepts that are frequently associated to suspenseful development or threatening outcome situations. However, the amount of ANEW terms that are potentially related to suspense is low (specifically from the middle-high terms sorted by decreasing valence). These tendencies have also been found in previous studies (Lazarus, 1966; Comisky and Bryant, 1982; Guidry, 2005; Madrigal et al., 2011; Lehne and Koelsch, 2015).

The potential causes and effects of these distributions will be discussed further.

### 3.2. Associations Between Affective Dimensions

In this section, comparisons between the different emotional dimensions are conducted through the corresponding regression analyses, taking the affective valence as the independent factor in line with the works of Redondo et al. (2007), Ferré et al. (2012), Soares et al. (2012), Monnier and Syssau (2014), and Hinojosa et al. (2016). Additionally, the words with the highest and lowest scores are studied, similarly to the previous valence dimension case.

#### 3.2.1. Valence vs. Arousal Dimensions

**Figure 3:**
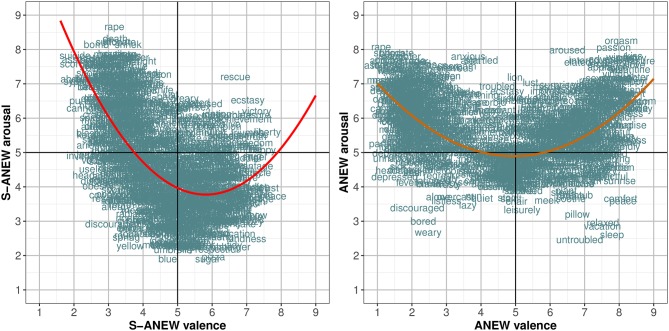
Distribution of the mean values corresponding to the suspense ratings of the 1,034 words in the dimensions of valence and arousal.

Figure 3 shows the ratings for the 1,034 words in the two-dimensional affective spaces corresponding to valence and arousal.

In both datasets, a significant U-shaped quadratic correlation between valence and arousal was found. The Spanish S-ANEW correlation is given by the formula *a* = 0.285*v*^2^−3.322*v*+13.441 [*R*^2^ = 0.361, *F*_(2, 1031)_ = 293.4, *p* < 0.0001])^5^, and represents 36.12% of the variance. This is similar to the results obtained by other authors as Moltó et al. (1999) against the 27.14% found in Redondo et al. (2007) ANEW and given by the formula *a* = 0.137*v*^2^−1.349*v*+8.227 [*R*^2^ = 0.271, *F*_(2, 1031)_ = 193.4, *p* < 0.0001]. The ratings distribution between valence and arousal showed that the terms rated as either strongly pleasant or strongly unpleasant are also consistently rated as more arousing. Also, in Spanish S-ANEW there is a predominance of unpleasant words that are rated as more arousing than pleasant words. Even though this asymmetry had not been clearly identified in the work of Redondo et al. (2007), similar distributions were found in other previous studies (Ferré et al., 2012; Montefinese et al., 2014; Soares et al., 2012; Guasch et al., 2016). Figure 3 (left) shows that most of the words with valence greater than 5 are located in the lower right of the chart, presenting medium to low values along the arousal dimension.

An analysis of the arousal scores along the ranges of valence (negative, neutral, and positive) supports the observed asymmetry in Spanish S-ANEW [χ^2^ = 330.02, *F*_(2, 1031)_ = 260.60, *p* < 0.0001]. Nevertheless, a *post-hoc* analysis only revealed significant arousal differences for the negative words, but not for neutral and positive words.

In order to find any tendencies in the highest and lowest arousal-rated words, two samples with the thirty highest rated terms per classification of both negative and positive valence were gathered from Spanish S-ANEW and Redondo et al. (2007) ANEW datasets. Due to the shape of the curve, it was necessary to check how the highest arousal-rated words differ between the extremes of the valence. Additionally, other sample of the thirty lowest rated terms was analyzed.

Table 4 shows the set of negative words (left) and positive words (right) evoking the highest arousal for both Spanish S-ANEW and Redondo et al. (2007) ANEW datasets. Regarding the negative words, the ten words shared by the two datasets (33.33%) are highlighted. Four of these terms were in the first positions of both lists, almost coinciding in order (with the exception of *drown*) and all of them were outliers in the distribution of the Spanish S-ANEW arousal ratings. According to the range of scores (see Table 2 and Figure 2), Spanish S-ANEW arousal ratings were slightly higher than Redondo et al. (2007) ANEW arousal ratings, unlike valence ratings, which behaved conversely. In addition, an analysis of the words revealed that most of Spanish S-ANEW terms referred to concepts either related to tragic physical effects (40.00%) (*death, rape, drown, suffocate, mutilate, paralysis, crushed, mangle, dead, torture, scorching, cancer*), related to potential threats (33.33%) (*bomb, massacre, outrage, killer, terrorist, assassin, danger, fire, sour, beast*), or, in a lower proportion, related to emotional states (10.00%) (*horror, panic, fear*). In comparison, Redondo et al. (2007) ANEW list contained appreciably less concepts related to tragic physical effects (20.00%) (*rape, suffocate, drown, torture, abuse*), and a similar number of terms related to potential threats (33.33%) (*bomb, slaughter, accident, danger, shark, terrorist, robber, tragedy, war, avalanche*), but more focused on emotional states (30.00%) (*panic, anxious, alert, rage, enraged, rabies, nervous, stress, despairing*). Other words such as physical locations (*morgue, ambulance*) or environment features (*dark*) were also presented in one or both lists, although in a smaller proportion. The classification of valence for most of the words was shared between the two datasets, with the exception of *fire* (neutral for Redondo et al., 2007 ANEW), *anxious* and *ambulance* (both neutral for Spanish S-ANEW).

**Table 4:** The thirty negative valence (left) and positive valence (right) highest arousal rated words.

**Highest arousal negative words**						**Highest arousal positive words**					
**S-ANEW**	**val**	**aro**	**ANEW**	**val**	**aro**	**S-ANEW**	**val**	**aro**	**ANEW**	**val**	**aro**
***Rape***	3.17	8.56	***Rape***	1.11	7.98	***Rescue***	6.67	7.11	***Orgasm***	8.06	8.16
Death	3.19	8.21	***Suffocate***	1.48	7.80	Ecstasy^•^	7.03	6.43	Passion	7.84	7.96
***Drown***	3.22	8.14	***Bomb***	1.42	7.79	***Victory***	7.29	6.11	Aroused^•^	6.50	7.89
***Suffocate***	3.22	8.12	***Panic***	1.53	7.72	Option	6.17	6.02	Kiss	8.43	7.71
***Bomb***	2.65	8.04	Anxious^•^	3.63	7.70	***Pleasure***	7.13	6.00	Fun	8.32	7.68
Shriek	3.57	8.04	Slaughter	1.66	7.70	Masturbate^•^	6.41	5.99	***Win***	7.84	7.68
Horror	3.08	7.77	***Drown***	1.32	7.61	***Chance***	6.03	5.95	Merry	8.41	7.66
Massacre	3.19	7.76	Accident	1.32	7.58	***Achievement***	6.92	5.88	Party	8.26	7.66
Mutilate	3.29	7.76	Alert	3.69	7.58	Alive	6.53	5.73	Adventure^•^	7.76	7.62
Suicide	2.03	7.72	Rage	2.11	7.58	Champion	6.22	5.66	Sex^•^	7.76	7.59
Paralysis	3.39	7.71	***Danger***	2.02	7.56	Liberty	7.59	5.50	Intercourse^•^	7.26	7.58
Outrage	3.36	7.69	***Killer***	1.23	7.49	***Triumph***	7.02	5.48	***Pleasure***	8.48	7.57
***Panic***	3.41	7.68	Enraged	2.02	7.48	***Win***	7.11	5.43	Elated^•^	6.80	7.53
***Killer***	3.88	7.67	Rabies	2.33	7.47	Sunrise	7.16	5.42	Excitement^•^	7.86	7.49
Crushed	2.71	7.67	***Torture***	1.24	7.47	Success	6.39	5.40	Erotic	7.44	7.47
Mangle	3.25	7.67	Ambulance^•^	2.34	7.44	***Orgasm***	6.37	5.40	***Love***	8.50	7.46
***Terrorist***	3.33	7.67	***Assassin***	1.18	7.44	Mind	6.32	5.37	Applause^•^	7.67	7.39
Morgue	3.20	7.63	Scream	3.39	7.42	Truth	7.28	5.37	Travel	7.84	7.39
Dead	2.76	7.63	Abuse	1.39	7.39	Loved	6.67	5.33	Valentine	8.33	7.32
***Assassin***	2.21	7.62	Shark	3.07	7.38	Bliss	6.22	5.31	Wish^•^	7.98	7.31
Betray	2.57	7.60	Nervous	3.26	7.36	Army^−^	6.05	5.21	***Victory***	7.93	7.19
Dark	3.36	7.57	Anger	2.50	7.33	Hope	6.40	5.20	Laughter	8.34	7.10
Fear	3.24	7.56	***Terrorist***	1.51	7.33	Freedom	7.23	5.18	Couple^•^	7.91	7.08
***Danger***	3.46	7.55	Robber	2.02	7.32	Triumphant	6.36	5.14	***Chance***	7.91	7.07
Fire^•^	3.58	7.53	Stress	1.83	7.32	Free	6.25	5.13	Romantic	7.99	7.07
Sour	2.61	7.51	Nightmare	1.80	7.31	Wit	6.94	5.13	Sexy^•^	7.33	7.07
***Torture***	2.69	7.50	War	1.23	7.28	Vigorous	6.02	5.09	***Triumph***	7.89	7.03
Scorching	2.21	7.48	Despairing	2.00	7.27	***Love***	7.07	5.07	***Rescue***	7.27	7.00
Beast	3.11	7.48	Tragedy	1.64	7.24	Intellect	6.96	5.07	***Achievement***	8.01	6.99
Cancer	3.24	7.46	Avalanche	2.46	7.20	Profit	6.33	5.04	Happy	8.37	6.97

Existing words in both dataset are remarked.

^+^, positive;

•, *neutral; or ^−^, negative word in the other dataset*.

In the other extreme of the range of values for valence, Table 4 (right) compares the set of positive words that scored the highest for arousal. In contrast to the list of negative words, the list of the highest arousal Spanish S-ANEW positive words contained terms related to chances or means to control a situation (26.67%) (*option, chance, mind, army, hope, wit, vigorous, intellect*), semantically related to the achievement of an objective or succeeding in general (30.00%) (*victory, achievement, champion, triumph, win, success, bliss, triumphant, profit*), and, more specifically, related to outcomes that imply escape (16.67%) (*rescue, alive, liberty, freedom, free*). In the case of Redondo et al. (2007) ANEW ratings, terms related to the achievement of an objective or escaping were also present to a lesser extent (20.00%) (*win, victory, chance, triumph, rescue, achievement*), and most of the remaining words were related to love and sexual themes (46.66%) (*orgasm, passion, aroused, kiss, sex, intercourse, pleasure*^6^, *excitement, erotic, love, valentine, couple, romantic, sexy*) and generic pleasant activities (30.00%) (*fun, merry, party, adventure, elated, applause, travel, laughter, happy*). In this regard, almost half of the words related to sexual issues and appearing in the Spanish S-ANEW list were classified as neutral in Spanish S-ANEW dataset.

Finally, Table 5 compares the set of words considered to evoke the least arousal, respectively, for both Spanish S-ANEW or Redondo et al. (2007) ANEW datasets. In this case, no words were shared between the resulting lists. As illustrated in Figure 3 (left), the lowest arousal Spanish S-ANEW words were mostly located in the neutral part of the valence range ([3.63, 6.65]), and corresponded to Redondo et al., 2007 ANEW neutral and positive valence words. Several of these Spanish S-ANEW terms were related to food (36.67%) (*sugar, pizza, jelly, milk, chocolate, hamburger, mushroom, muffin, butter, ketchup, salad*). On the other hand, the Redondo et al. (2007) ANEW list presented a wider range for valence ([2.16, 8.11]) and a lower mean arousal. In the Redondo et al. (2007) ANEW list, the meaning of most of the terms is related to non-stress and well-being.

**Table 5:** The thirty lowest arousal rated words.

**S-ANEW**	**val**	**aro**	**ANEW**	**val**	**aro**
Blue^+^	4.72	1.80	Untroubled^•^	6.86	2.36
Sugar^+^	5.87	1.81	Sleep^•^	7.79	2.51
Pizza^+^	6.04	1.86	Weary^−^	2.47	2.58
Umbrella^•^	4.87	2.07	Vacation^•^	7.52	2.76
Respectful^+^	6.13	2.09	Relaxed^+^	7.53	2.86
Jelly^•^	5.22	2.12	Bored^−^	2.33	2.90
Milk^•^	5.22	2.14	Pillow^•^	6.79	3.10
Chocolate^+^	5.97	2.15	Leisurely^•^	5.19	3.23
Hamburger^•^	6.41	2.16	Discouraged^−^	2.16	3.29
Tennis^•^	5.38	2.16	Lazy^−^	3.62	3.37
Dustpan^•^	5.16	2.16	Chair^•^	5.03	3.48
Dancer^+^	5.35	2.17	Listless^−^	3.00	3.49
Yellow^•^	3.63	2.18	Peace^+^	8.11	3.49
Circle^•^	4.58	2.19	Meek^+^	5.90	3.51
Athletics^+^	5.09	2.19	Soothe^+^	6.60	3.56
Name^•^	5.33	2.20	Comfort^+^	8.04	3.56
Tidy^+^	6.65	2.20	Saint^•^	4.87	3.56
Mushroom^•^	4.66	2.21	Quiet^•^	4.03	3.57
Muffin^+^	5.49	2.21	Stool^•^	4.79	3.59
Butter^•^	4.64	2.21	Overcast^−^	3.27	3.61
Dollar^•^	5.73	2.21	Alone^−^	2.76	3.61
Ketchup^•^	5.27	2.21	Dream^−^	6.60	3.63
Silk^+^	4.90	2.22	Nun^•^	3.79	3.64
Vest^•^	5.32	2.23	Bathtub^•^	6.76	3.66
Yacht^+^	5.34	2.23	Stove^•^	6.37	3.74
Salad^+^	4.54	2.24	Basket^•^	5.18	3.79
Kerchief^•^	4.69	2.27	Subdued^−^	5.23	3.79
Frog^•^	5.13	2.27	Plant^•^	6.46	3.80
Butterfly^+^	5.53	2.27	Plain^•^	5.18	3.84
Sissy^•^	4.69	2.28	Bowl^•^	5.01	3.89

Existing words in both dataset are remarked.

+, positive;

•, neutral; or

−, *negative word in the other dataset*.

#### 3.2.2. Valence vs. Dominance Dimensions

**Figure 4:**
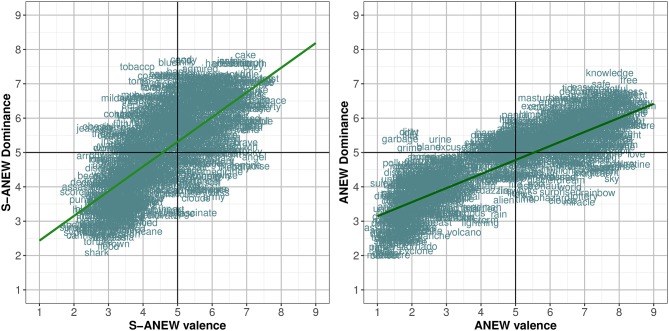
Distribution of the mean values corresponding to the suspense ratings of the 1,034 words in the dimensions of valence and dominance.

Figure 4 shows the ratings for the 1,034 words in the two-dimensional affective spaces corresponding to valence and dominance.

Dominance (*d*) had a positive correlation with valence (*v*) in Spanish S-ANEW [*r* = 0.697, *F*_(1, 1032)_ = 977.8, *p* < 0.0001], following the formula *d* = 0.719*v*+1.715 and presenting a higher variation than the one found in Redondo et al. (2007) ANEW [*r* = 0.827, *F*_(1, 1032)_ = 2241, *p* < 0.0001]. The relationship was also linear, implying that higher dominance ratings were assigned to words associated with pleasant concepts. These results were consistent with the findings of authors who analyzed both dimensions (Montefinese et al., 2014; Warriner et al., 2013; Moors et al., 2013).

Once again, the study of this distribution was extended through the analysis of the highest and lowest rated words. Table 6 shows the set of words with the lowest and highest ratings for dominance, thirty for each dataset. All the lowest rated words were in the range of negative valence. They were also rated as unpleasant in the other dataset. As with the lowest valence-rated words, the lowest rated terms were mostly related to individual or large-scale tragedies (either cause or effect), without specific semantic groups that clearly differentiated Spanish S-ANEW from Redondo et al. (2007) ANEW. Eight words (26.67%) were shared across both sets.

**Table 6:** The thirty lowest (left) and highest (right) dominance rated words.

**Lowest dominance**						**Highest dominance**					
**S-ANEW**	**val**	**dom**	**ANEW**	**val**	**dom**	**S-ANEW**	**val**	**dom**	**ANEW**	**val**	**dom**
Shark^−^	2.66	1.99	Cancer^−^	1.23	1.91	Cake^+^	6.97	7.75	Knowledge^+^	7.73	7.22
***Flood***^−^	3.00	2.17	Massacre^−^	1.32	1.92	Candy^+^	5.17	7.61	Free^+^	8.28	7.01
***Drown***^−^	3.22	2.24	Death^−^	1.23	1.93	God^•^	5.17	7.59	***Safe***^+^	7.48	6.90
***Torture***^−^	2.69	2.31	***Dead***^−^	1.17	1.98	Inspire^+^	6.57	7.57	Easy^+^	6.92	6.80
Devil^−^	2.84	2.43	***Tumor***^−^	1.34	1.98	***Safe***^+^	6.48	7.57	Tidy^+^	6.57	6.78
Helpless^−^	2.99	2.44	***Cyclone***^−^	2.13	2.04	Milk^•^	5.22	7.53	Respectful^+^	7.63	6.76
Trauma^−^	3.29	2.44	War^−^	1.23	2.04	Blue^+^	4.72	7.52	Capable^•^	7.52	6.70
Cannon^−^	2.26	2.49	Rape^−^	1.11	2.13	Triumph^+^	7.02	7.52	Improve^+^	7.46	6.69
***Tumor***^−^	3.16	2.50	Killer^−^	1.23	2.16	Handsome^+^	6.48	7.50	Caress^+^	8.27	6.68
Depression^−^	3.21	2.52	Paralysis^−^	1.33	2.16	***Education***^+^	6.63	7.50	Decorate^•^	6.83	6.68
Assault^−^	2.70	2.57	Tornado^−^	2.24	2.17	Truth^+^	7.28	7.49	Comfort^+^	8.04	6.63
Syphilis^−^	2.17	2.58	***Bomb***^−^	1.42	2.22	Cozy^+^	7.19	7.40	Music^•^	8.16	6.61
***Bomb***^−^	2.65	2.61	Terrorist^−^	1.51	2.28	Tobacco^−^	3.83	7.40	Bath^•^	7.21	6.59
Hurricane^−^	3.96	2.61	Nightmare^−^	1.80	2.30	Admired^+^	5.65	7.34	Graduate^•^	7.09	6.59
Kerosene^−^	2.68	2.61	Suffocate^−^	1.48	2.33	Basket^•^	5.12	7.26	Satisfied^•^	7.88	6.59
Volcano^−^	2.72	2.61	Robber^−^	2.02	2.34	Nectar^+^	5.50	7.20	Achievement^+^	8.01	6.58
***Panic***^−^	3.41	2.65	Abduction^−^	1.67	2.36	Banner^•^	4.63	7.19	Vacation^•^	7.52	6.54
Funeral^−^	2.50	2.67	Slaughter^−^	1.66	2.39	Cuddle^+^	6.93	7.18	Good^+^	7.92	6.53
Embattled^−^	3.44	2.68	Burial^−^	1.36	2.40	Beautiful^+^	6.57	7.17	Alive^+^	8.07	6.53
Jail^−^	3.34	2.68	***Drown***^−^	1.32	2.41	Sky^+^	6.24	7.17	Masturbate^+^	5.79	6.49
Suicide^−^	2.03	2.72	***Panic***^−^	1.53	2.43	Meek^•^	6.20	7.17	***Education***^+^	7.07	6.48
Invader^−^	2.25	2.73	Sickness^−^	1.51	2.44	Chin^•^	4.61	7.15	Honor^+^	7.36	6.47
***Dead***^−^	2.76	2.74	***Torture***^−^	1.24	2.44	Coarse^−^	4.28	7.14	Intellect^+^	6.56	6.47
Smallpox^−^	2.81	2.74	Avalanche^−^	2.46	2.48	Diploma^+^	4.75	7.14	Victory^+^	7.93	6.47
Slave^−^	2.94	2.74	Funeral^−^	1.48	2.50	Dustpan^•^	5.16	7.14	Idea^•^	7.36	6.46
***Cyclone***^−^	3.05	2.75	Ulcer^−^	1.70	2.51	Glamor^•^	6.25	7.13	Song^•^	8.01	6.44
Mutilate^−^	3.29	2.76	***Flood***^−^	1.99	2.52	Hotel^+^	5.20	7.12	Happy^+^	8.37	6.44
***Tragedy***^−^	3.30	2.77	***Tragedy***^−^	1.64	2.52	Bouquet^+^	5.41	7.12	Prestige^•^	7.17	6.44
Cliff^−^	3.59	2.78	Destruction^−^	1.50	2.53	Acceptance^+^	6.26	7.10	Confident^•^	6.51	6.43
Crushed^−^	2.71	2.78	Corpse^−^	1.41	2.54	Limber^•^	5.41	7.09	Salute^•^	6.86	6.42

Existing words in both dataset are remarked.

+ , positive;

•, neutral; or

−, *negative word in the other dataset*.

Regarding the highest rated dominance and as shown in Figure 4, most of Spanish S-ANEW rated words lied within the range of neutral valence, while most of Redondo et al. (2007) ANEW rated words were within the range of positive valence. Beyond that, no significant semantic commonalities were found.

As expected, in Spanish S-ANEW, arousal and dominance have a similar correlation to the one found between valence and arousal [$R^{2}=0.441,F_{\left(2,1031\right)}=408.2,p<0.0001$]. This behavior is in line with the findings from other studies (Warriner et al., 2013; Montefinese et al., 2014). The curve follows the formula *a* = 0.119*d*^2^−1.918*d*+11.054.

#### 3.2.3. Interpretation of Results

The study of the inter-dependencies between valence and arousal shows that subjects' rate as more arousing either strongly pleasant or strongly unpleasant words, regardless of the context. However, when a suspenseful context is introduced, unpleasant words are rated with a higher arousal than pleasant words are. It could be said that suspense diminishes arousal for the most pleasant concepts. This might be related to the lower amount of words ranked with a positive valence in a suspenseful scene. In a suspenseful context, we found that there are roughly twice as much negative valence words as positive valence words. Also, Spanish S-ANEW has half the words with positive valence that Spanish S-ANEW has. Since suspenseful scenes are usually conceived as unpleasant, it makes sense to assume that less involved concepts are perceived as positive.

Moreover, in a context of suspense, subjects assign the highest arousal to negative concepts related to tragic physical effects and potential threats. Likewise, positive concepts related to control a situation, and again to achieve an objective or escaping a threat were rated with the highest arousal as well. By contrast, when no context is present, the list included less words related to tragic physical effects, but more negative concepts involving emotional states, as well as positive concepts linked to interpersonal relations, were rated with the highest values for arousal.

Regarding the dominance dimension, a linear high correlation with valence implies that subjects usually feel in control when facing pleasant concepts and vice-versa, although to a lesser degree in a context of suspense. Thus, in this context the valence impact is significant but not as determinant as to evoke a sense of control. In any case and even considering that the average dominance is lower with the suspense backdrop (as explained in the previous sections), the similarities between ratings suggest that the words elicit proportionally similar emotional reactions to the feeling of dominance regardless of the context.

Furthermore, there are no different semantic groups between both the highest and the lowest dominance rated words. Regarding the lowest rated concepts, both suspense contextualized and non-contextualized sets share around 25% of words. All of them were considered unpleasant and most of them were related to individual or large-scale tragedies.

In conclusion, while both datasets do not present differences for dominance, the ratings and the semantics substantially differ for arousal. On the one hand, in a context of suspense, unpleasant words evoke significantly less arousal than pleasant words. On the other hand, words associated with certain semantic themes like tragic physical effects, potential threats, control, or escaping, tend to be rated with the highest arousal mainly or only in the context of suspense.

### 3.3. Gender Differences

For each emotional dimension and term, subjects' responses were analyzed in terms of gender-related variations. Table 7 presents means, standard deviations, range values, correlation indexes, and differences of means of the Spanish S-ANEW ratings for both females and males in the three affective dimensions, globally and per valence classification.

**Table 7:** Means, standard deviations, range values, correlation indexes and differences of means of the 1,034 words ratings of the Spanish S-ANEW for females and males in the three affective dimensions, globally and per valence classification.

	**Word**	**Male**		**Female**		
**Dim**.	**Type**	**Mean (SD)**	**Range**	**Mean (SD)**	**Range**	**R**
val	(Global)	4.60 (1.08)	[2.07, 7.63]	4.67 (1.49)	[1.96, 7.93]	0.93
	Negative	3.51 (0.44)	[2.07, 4.45]	3.09 (0.48)	[1.96, 4.42]	0.61
	Neutral	4.86 (0.51)	[3.73, 6.04]	5.12 (0.74)	[3.34, 6.90]	0.64
	Positive	6.29 (0.43)	[5.43, 7.63]	6.91 (0.45)	[5.88, 7.93]	0.61
aro	(Global)	4.59 (1.33)	[1.85, 8.48]	4.63 (1.60)	[1.71, 8.69]	0.93
	Negative	5.61 (1.20)	[2.38, 8.48]	5.78 (1.50)	[1.88, 8.69]	0.92
	Neutral	4.01 (1.04)	[1.85, 7.42]	3.89 (1.25)	[1.71, 7.78]	0.89
	Positive	3.95 (0.87)	[1.93, 6.88]	4.16 (1.20)	[1.77, 7.45]	0.87
dom	(Global)	5.03 (1.13)	[2.06, 7.67]	5.06 (1.52)	[1.77, 7.96]	0.91
	Negative	4.08 (0.86)	[2.06, 7.21]	3.79 (1.17)	[1.77, 7.67]	0.84
	Neutral	5.43 (0.86)	[3.05, 7.60]	5.60 (1.18)	[2.64, 7.96]	0.84
	Positive	6.01 (0.76)	[3.84, 7.67]	6.37 (0.99)	[2.82, 7.87]	0.82

*All values are significant (p < 0.001)*.

Although scores were highly similar between males and females, when comparing the ratings according to their valence classification, females presented greater average ratings than males for positive words and lower than males for negative words. Also, the range of scores was higher for female subjects in the three dimensions. These results are in line with Monnier and Syssau (2014) and Soares et al. (2012), who found that ratings for affective stimuli were lower for males than for females. Additionally, strong correlations where found between men and women ratings across all dimensions (*R*> = 0.91, *p* < 0.0001), which is broadly supported by most of studies (Bradley and Lang, 1999; Redondo et al., 2007; Monnier and Syssau, 2014; Montefinese et al., 2014; Soares et al., 2012; Lang et al., 1997).

In order to study other possible dependencies for different combinations of gender, dimension and valence, a multivariate analysis MANOVA was conducted. First, a global, marginal effect in gender was found [*F*_(1, 6202)_ = 3.196, *p* = 0.073]. Second, a significant interaction between *gender* × *dimension* × *valence classification* was also observed [*F*_(4, 6199)_) = 16.452, *p* < 0.0001]. *Post-hoc* tests indicated that valence ratings (*p* < 0.01 for all the valence classifications), and dominance in the range of negative valence (*p* < 0.01) differed significantly depending of the gender of the subject. This result does not coincide with Redondo et al. (2007) findings, in which sex did not reach statistical significance, beyond female subjects tended to rate negative words as more negatively and vice-versa. However, results were mostly consistent with other works, such as the original research of Bradley and Lang (1999), who obtained statistically significant differences for the dominance dimension, or Soares et al. (2012), who found that females tend to assign higher ratings to pleasant words.

**Figure 5:**
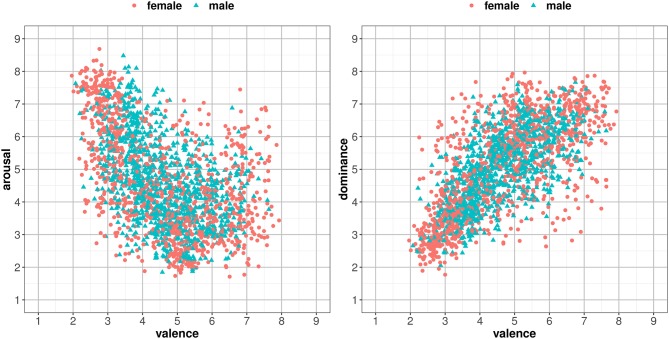
Distributions of the 1,034 Spanish S-ANEW word ratings in the affective space defined by *valence*, and *arousal* **(Left)** and *dominance* **(Right)**, for both males and females.

Regarding the association between dimensions, Figure 5 shows the distribution of the Spanish S-ANEW scores in the affective space defined, respectively, by valence and arousal, and by valence and dominance, filtering male and female ratings. These dependencies showed significant differences for arousal, fitting female subjects' scores (*R* = 0.640, *p* < 0.0001) better than male subjects' scores (*R* = 0.534, *p* < 0.0001). By contrast, similar correlation values for dominance, both for females (*R* = 0.690, *p* < 0.0001) and males (*R* = 0.677, *p* < 0.0001), were found.

#### 3.3.1. Interpretation of Results

Results suggest that response to emotional stimuli is lower for males than females in valence and positive dominance ratings. Likewise, considering the U-shape curve that relates arousal and valence, women rate with a higher arousal value the words with either low or high valence, while men seem less affected by the words in the upper and lower limits of the valence range. These observations do not coincide with the findings of Redondo et al. (2007) or Monnier and Syssau (2014), for whom there are not significant differences related to gender. However, a number of Redondo et al. (2007) ANEW studies also support gender-related variations in one or more dimensions (Bradley and Lang, 1999; Soares et al., 2012; Stevenson et al., 2007). Thus, there is no agreement regarding the impact of the gender in the affective evaluations in the literature (Soares et al., 2012; Montefinese et al., 2014; Redondo et al., 2007), so at this point it is not possible to assure that the context is a relevant factor for gender-related variations in the results.

### 3.4. Comparison With Scores for Suspense

Once analyzed, Spanish S-ANEW ratings were contrasted with the suspense scores gathered in Delatorre et al. (2017). This corpus was composed by twenty-five words. Each one of them was introduced in two different suspenseful scenes: a short text passage and an interactive 3D environment. Table 8 shows the words and the scores.

**Table 8:** Means and medians of suspense perceptions for each object.

	**S-ANEW**			**ANEW**			**Suspense**	
**Word**	**val**	**aro**	**dom**	**val**	**aro**	**dom**	**Text**	**3D**
Corpse	3.30	7.13	3.15	1.41	6.87	2.54	7.02	7.24
Penalty	3.76	3.92	4.52	1.98	6.67	3.62	2.76	2.18
Germs	3.08	4.64	4.06	1.78	6.26	3.42	3.57	6.12
Vomit	3.11	4.73	3.57	1.76	5.80	3.12	4.94	5.06
Manure	4.03	4.06	4.03	2.02	4.64	4.40	3.41	6.18
Dirt	3.81	4.33	3.52	1.98	5.61	5.48	3.40	5.18
Mucus	3.79	4.13	6.58	2.58	4.69	4.08	4.03	4.88
Skull	3.42	6.80	4.70	4.42	4.94	4.63	5.76	5.12
Thermometer	4.72	3.31	5.94	4.28	4.49	4.87	2.54	1.69
Dummy	3.03	5.46	5.03	4.38	3.91	4.99	5.38	5.35
Flag	4.71	3.59	5.24	4.92	4.36	5.04	2.44	3.41
Computer	5.82	5.47	6.90	5.50	5.06	5.27	3.13	2.59
Hat	5.02	3.01	6.25	4.87	4.11	5.46	2.89	1.59
Bowl	5.56	3.39	6.45	5.01	3.89	5.34	2.25	1.41
Jug	5.97	2.85	6.61	4.81	4.17	5.83	2.31	1.65
Money	6.12	3.80	5.39	7.46	6.40	5.50	3.10	2.06
Sugar	6.87	1.81	6.83	6.68	5.59	5.67	2.05	1.29
Honey	5.47	4.74	6.56	5.46	4.06	5.66	2.10	1.71
Dress	5.22	4.07	5.53	6.32	5.32	6.16	4.13	2.88
Diploma	4.75	2.33	7.14	7.46	6.10	6.01	2.18	2.59
Chocolate	5.96	2.15	6.93	7.72	6.10	5.84	2.25	1.71
Doll	4.75	4.72	5.34	6.24	4.48	5.79	5.44	3.29
Muffin	5.49	2.21	7.09	6.27	4.26	5.64	2.10	1.24
Flower	5.36	2.44	6.36	7.34	4.46	5.69	2.56	1.82
Bed	6.64	3.71	6.36	7.71	4.57	6.02	3.38	2.65

In order to determine the relationship between suspense and the affective dimensions, a multiple regression analysis was conducted with the suspense mean ratings as the dependent factor, while both Redondo et al. (2007) ANEW and Spanish S-ANEW ratings were used as independent factors. The resulting models were compared to determine the best fit.

We performed separate analyses for each dataset by combining the three dimensional variables [valence (*v*), arousal (*a*), and dominance (*d*)], to predict the suspense ratings in a similar way as existing analyses found in the literature (Montefinese et al., 2014; Stevenson et al., 2007; Riegel et al., 2016; Jacobs et al., 2015). The formula of the best-fit model for Spanish S-ANEW dimensions [*R* = 0.844, *F*_(3, 47)_ = 39.44, *p* < 0.0001, *RMSE* = 0.860] included the three variables. However, the valence ratings were barely significant (*t* = −1.35, *p* = 0.187), and excluding it did not imply a substantially worse fit (*p* = 0.184). Thus, only arousal and dominance were ultimately included. The new model presented a similar high adjustment [*R* = 0.841, *F*_(2, 47)_ = 57.95, *p* < 0.0001, *RMSE* = 0.868], explaining 70.8% of the variance. The obtained formula for this model was 0.533*a*−0.622*d*+4.636. Moreover, other polynomial and linear estimations did not result in significant improvements.

**Figure 6:**
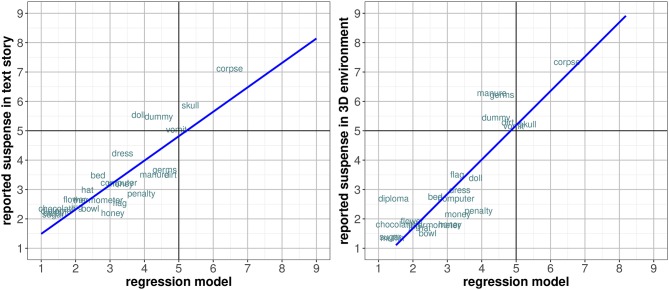
Comparison of correlation between Spanish S-ANEW regression model and the reported suspense in the textual story **(Left)** and the interactive 3D environment **(Right)**.

The model was strongly correlated to the reported suspense, for both the textual story (*r* = 0.878, *p* < 0.0001) and the interactive 3D environment (*r* = 0.834, *p* < 0.0001). Figure 6 illustrates this.

Regarding Redondo et al. (2007) ANEW ratings, a lineal regression that included only dominance was the best-fit model [*R* = 0.606, *F*_(1, 47)_ = 27.66, *p* < 0.0001, *RMSE* = 1.283], accounting for 36.2% of the variance. The obtained formula was −1.008*d*+8.395. The model was medium correlated to the suspense ratings for the text story (*r* = 0.534, *p* < 0.0005), and medium to strongly correlated for the interactive 3D environment (*r* = 0.685, *p* < 0.0005). These values were clearly lower in comparison to the model computed for Spanish S-ANEW affective ratings.

#### 3.4.1. Interpretation of Results

Valence as negative factor positively contributes to a small better adjustment, meaning that unpleasant words have an impact in suspense responses. Actually, some authors specialized on suspense have included concepts as “danger,” “hostile,” “deplorable,” or “harmful” in their definitions (Perron, 2004; Zillmann and Tannenbaum, 1980; Zillmann, 1996; de Wied et al., 1992). Likewise, words that evoke similar ideas rated the lowest in terms of valence (see Table 3). Despite of this, when analyzing the statistical impact of valence in the model, it seems not to improve the adjustment significantly. This observation is in line with other modern definitions that avoid explicitly giving a negative connotation to the causes that trigger suspense, but only to the emotional responses derived from suspense (fear, frustration, anxiety, concern, apprehension) (Abbott, 2008; Alwitt, 2002; Vorderer and Knobloch, 2000; Smuts, 2008; Caplin and Leahy, 2001; Guidry, 2005; Knobloch et al., 2004). Considering the general conception of suspense as a feeling of anticipation (Wang and Cheong, 2006; Burget, 2014; Wirth and Schramm, 2005; Lehne, 2014), all those definitions seem to agree that the outcome must just bring “significant consequences (either good or bad) (Brewer and Lichtenstein, 1982), being anything that potentially causes positive or negative future changes (the promise of a kiss, a pay rise, a loose bolt in the airplane's wheel) (Howard, 2006; Smuts, 2008; Burget, 2014). Therefore, although it is not possible to discard at all the impact of the negative valence (and, in fact, the number words rated as positive substantially decreases in a suspenseful context, as shown in Table 2), its degree of influence is put into question according to both the literature on the topic and the statistical result, that is barely determinant at least in contrast to the other affective dimensions.

Arousal, on the other hand, fits the model better. The most unpleasant and the most pleasant words get the highest ratings of arousal, which presents a reduced dependency on the polarity of the valence. Moreover, the highest arousal ratings in the context of suspense comprise a substantial proportion of specific concepts related to potential threats and tragic physical effects, as well as (in contrast to Redondo et al., 2007 ANEW scores) controlling a situation, achieving an objective, and escaping a threat, concepts that may also be related to being in control. Along with this, large-scale tragedies have been rated mostly with the lowest dominance. Despite the fact that these terms can be found in a diversity of contexts, they may be often observed working together specifically in generating suspense (Allen and Ishii-Gonzales, 2004; Gerrig and Bernardo, 1994; Frome and Smuts, 2004; Truffaut and Scott, 1998). These observations provide evidence in favor of arousal and dominance having a strong relation to the emotional response, terms and chain of events which generally characterize a suspenseful scene (Hsu et al., 2014).

On the other hand, the regression model for Redondo et al. (2007) ANEW scores presents a worse adjustment. Besides, its accuracy seems to depend on the narrative medium, as it does not fit the text story's suspense equally to the interactive 3D environment's. However, the general behavior of the individual curves of both arousal and dominance ratings is not too different from ones found in Spanish S-ANEW as to explain these disparities. The more plausible assumption is that, even if the ratings are distributed in a similar way, the words included in each score range are highly influenced by the existence of the context. Indeed, the specific context of suspense has made it possible to obtain a set of affective ratings and a subsequent model that strongly fits the reported suspense.

### 3.5. Relation With Other Psycholinguistic Indices

The values for the affective dimensions of Spanish S-ANEW were compared to objective (number of letters, number of syllables, grammatical class, frequency, orthographic neighbors) and subjective psycholinguistic indices (familiarity, concreteness, imageability). Similarly, the reported suspense was compared with these variables to study potential relations, in both the textual story and the interactive 3D environment.

The results revealed very weak or no significant correlations for most of the candidate relations, with the exception of valence. This valence relations included a positive weak correlation with frequency (*r* = 0.154, *p* < 0.0001), and a positive weak to moderate correlation with familiarity (*r* = 0.201, *p* < 0.0001). Also, familiarity presented weak correlations with arousal (*r* = −0.165, *p* < 0.0001) and dominance (*r* = 0.183, *p* < 0.0001).

#### 3.5.1. Interpretation of Results

Considering the results of the familiarity subjective index, subjects seem to feel a significant low emotional bias when facing words that they either know or use more often. The more familiar the word is to them, the higher valence, the lower arousal, and the higher dominance is reported. This hypothesis is partially in line with the conclusion of Warriner et al. (2013) and Montefinese et al. (2014), although they found that this happens mainly for the dominance. They conclude that response is a “fear of the unknown,” which seems to be consistent with a state of anticipation and suspense.

Nevertheless, other correlations with subjective psycholinguistic indices found in relevant literature are not present enough in our results to include them as part of the affective responses to suspense. Since the current analysis was conducted by using the ratings gathered by Redondo et al. (2007), where no context was introduced, it would be necessary a new assessment of psycholinguistic indices in a specific context.

## 4. Discussion

Although these results represent an improvement over the previous models that predict suspense, some issues need to be discussed.

The proposed methodology has a number of limitations. First, in order to avoid restricting the context to a single kind of predetermined scene, no specific description was provided to the participants. This prevents the subjects from being forced to adapt the concepts for a previously manipulated scenario. It also reduces the risk of the participants not being able to come up with a coherent relation between the scene and some of the concepts, or consider some of the proposed scenes as not suspenseful. Therefore, it was decided to let the participants recreate and operate their own suspenseful scenes in a potentially natural way. This approach was inspired by similar procedures found in the literature, in which not even the term “suspense” is defined. These strategies assume the validity of the subjective criteria of the participants (Brewer and Lichtenstein, 1980; Comisky and Bryant, 1982; Gerrig and Bernardo, 1994; Hoeken and van Vliet, 2000; Alwitt, 2002; Cheong and Young, 2008; Abuhamdeh et al., 2015; Liang, 2015). As in the mentioned studies, this lack of definition implies an ambiguity in the method. In our case, a scene in a participant's mind can be different from one another. This can lead to different ratings for the same term, produced not only by the personal interpretation, but by the details of a different scene. This situation also occurs in the original ANEW studies (Bradley and Lang, 1999; Redondo et al., 2007; Soares et al., 2012; Moors et al., 2013; Warriner et al., 2013; Monnier and Syssau, 2014; Montefinese et al., 2014) where, even when no context is specified for each term, the represented concept will be placed in a specific context in the reader's mind once it is read (Citron et al., 2014a; Stanovich, 2017). This activation process seems to take place because the stimulus activates the memory, and therefore inter-subject divergences can happen even when the context is not previously set (Neumann, 1984; Stanovich, 2017). On this basis and due to the memory activation, we may assume a similar automated recall process when known and consistent contexts are required (Stanovich and West, 1979; Fazio, 2001; Rayner et al., 2012).

In any case, the similarity between the results among the previously mentioned suspense or ANEW experiments seems to indicate that this methodological ambiguity does not invalidate the quantification of the emotional effect of the terms. At the very least, it does not seem to be a discussed issue in the reviewed literature. Certainly, it is not possible to guarantee that the participants have interpreted or imagined the concepts in a context of suspense, but despite of this fact, we argue that the obtained model presents a better fit of the previous ratings of suspense than the original Redondo et al. (2007) ANEW data. This assumption, the different ratings of words with distinct semantic attribution, and the consistency in terms of reliability suggest that the contextualization has effectively happened, and that it can be quantified as described in the paper.

The second relevant aspect worth discussion concerns the procedure for gathering the results through a questionnaire. The methodology utilized to obtain the Spanish S-ANEW ratings is based on the one used in Moors et al. (2013), which differs from Redondo et al. (2007)'s methodology. Since this latter's dataset is the dataset used to contrast the Spanish S-ANEW scores, it might be argued that a similar method of assessment could ensure a more rigorous comparative analysis. Nevertheless, a number of experiments on gathering affective ratings (in which each experiment is also compared with each other) obtained assessment through strategies different from the classroom-based collective sessions used by Redondo et al. (2007) e.g., remote web surveys or spreadsheet (Montefinese et al., 2014; Hinojosa et al., 2016; Soares et al., 2012; Guasch et al., 2016; Moors et al., 2013), asking each participant for the evaluation of 56 (Montefinese et al., 2014) to 4,300 words (Moors et al., 2013). Ultimately, email or web-based methods are used by several authors due to the clear advantages in terms of time and resources (Buchanan and Smith, 1999; Reips, 2000; Risso, 2002). Regarding this, relevant literature points out very small differences between experiments in person and remote experiments, and in the psychometric properties, providing substantial similarities across all the results (see also Krantz et al., 1997; Smith and Leigh, 1997; Pasveer and Ellard, 1998; Stanton, 1998; Buchanan and Smith, 1999; Buchanan, 2000; Risso, 2002, among others). This suggests that the methodological differences are not significant enough to compromise the comparative analysis, nor to challenge the validity of the regression model. However, and as an issue worth mentioning, the lack of supervision makes it impossible to reliably control the number of participants' sessions and behaviors, or the time intervals, which can impact the task performance (Gasquet et al., 2001). Even though our collected data point toward a consistency in the results, it is acknowledged that there is a limitation in the experiments using remote questionnaires.

The comparative study carried out with the first and last thirty terms according to the rating and semantics of the words also deserves discussion. A number of semantic groups of terms for each dataset were identified by the authors. The objective was to illustrate the impact of the context in the ratings when considering the semantics. Due to the great variability of potential classifications, it is acknowledged that subjective interpretation can play an important role in the configuration of these semantic groups. Because no study has previously set any standard quantity, the specific value of thirty words (around 2.5% at each end) may be considered unjustified and potentially extensible. However, the observed words seem to be sufficient to defend that the context influences the ratings, which is illustrated by the positional order of the analyzed word when sorted by score.

This tendency indicates that, when contextualized and grouped by range (positive, neutral and negative), the scores seem to be higher for some specific semantically-related terms. This can be observed by focusing on the highest and lowest ranked terms on each affective dimension. For instance, we have observed that, in suspenseful contexts, subjects tend to assign higher scores to success-related terms in contrast to those implying interpersonal relations, since the latter have higher scores in uncontextualized scenarios. This tendency has also been described in the original ANEW (Bradley and Lang, 1999). Similar differences regarding context influence can be found in Tables 4–6.

In any case, the semantic study and the corresponding interpretation is intended to be discussed as one of the possible explanations for the model's improvement. Even when an increased number and a reinterpretation of the semantics could modify the observed groupings, the current range seems to evidence that some semantics are prioritized over others depending on the context. This is shown quantitatively in the referred tables. This ordinal and quantitative differentiation of the whole corpus from the compared ANEW is significant and large, as evidenced in the improvement (70% compared to the 36% of variance accounting) of the regression model obtained from the contextualized ratings when applied to the direct suspense ratings.

Finally, the set of suspense ratings in Delatorre et al. (2017) used to test the Spanish S-ANEW regression model, covers only a minor part of the original 1,034 words. The results may be considered robust enough: the same 25 concepts in two different narrative discourses have been contrasted with the model, explaining around 70% of the variance. However and despite of this evidence, it is not possible to discard the possibility that the model obtained might not appropriately predict suspense for others subsets from the original ANEW list, as well as any other narrative discourse. An experiment to gather an extensive set of scores will be addressed as part of the future work. Additionally, it should include new ratings for concreteness, imageability, context availability, and familiarity: It is conceivable that subjects face potential inconsistencies between the imagined scene and the rated word, and even that the onset of the word qualifies and alters the original background^7^. Other terms such as *theory, poetry, phase*, or *history* may be considered challenging to be literally integrated within the scene, so it is possible that subjects create a complex, more unique situation that associates these concepts. Gathering and analyzing subjective psycholinguistic indices should partially shed light on this bias.

## 5. Conclusions and Future Directions

The objective of this work is to provide quantifiable evidence for the impact of context on the ratings of affective norms, as opposed to the non-contextualized existing corpora. In order to do this, 206 Spanish subjects used the SAM model to evaluate valence, arousal and dominance of the 1,034 words proposed in Redondo et al. (2007) ANEW, in a fictional context of suspense. Both datasets were contrasted, revealing similar behavior in the curves of ratings for the three affective dimensions. However, on average, Spanish S-ANEW words tended to be less pleasant, significantly evoked less arousal, and presented a slightly higher dominance. An analysis of the highest and lowest rated words for the three dimensions reveals the existence of different semantic groups, such as the highest rated positive terms being related to escaping a threat for Spanish S-ANEW, in contrast with the highest rated positive terms being related to interpersonal relationships for Redondo et al. (2007) ANEW. This indicates that, when contextualized, groups categorized by range (positive, neutral and negative) tend to receive higher scores for some specific semantically-related terms. This can be detected by observing the highest and lowest affective ranking terms.

These results provide a quantification of a specific context influence on affective terms. Specifically, a general observation suggests that valence and dominance scores tend to be neutral to low for concepts that are expected to be present in a suspenseful scene. This difference may be explained by the effects of predictability on the readers (Illouz, 2009; Schacht and Sommer, 2009; Scott et al., 2012; Smith and Levy, 2013), which will be considered for a study in future contributions. In line with the mentioned literature (Lazarus, 1966; Boekaerts, 2001; Guidry, 2005; Carnaghi et al., 2008; Madrigal et al., 2011; Lehne and Koelsch, 2015).

Additionally and in order to validate the Spanish S-ANEW ratings, a multiple regression analysis was conducted against a dataset of suspense obtained from Delatorre et al. (2017), in which twenty-five elements introduced in both a short text and an interactive 3D environment were rated in terms of suspense. Once calculated, the model for Spanish S-ANEW obtained a strong correlation to suspense ratings (*r*≈0.85, *p* < 0.0001 in both narrative scenarios), while Redondo et al. (2007) ANEW regression model presented weaker correlations, as well as a high dependency on the media (*r* = 0.534, *p* < 0.0005 for the textual story; *r* = 0.685, *p* < 0.0005 for the interactive 3D environment). These findings support the validity of the Spanish S-ANEW corpus.

In conclusion, the result of this work provides an affective norms dataset in a specific context of suspense. We reckon this is an innovative proposal, as no other similar research that covers the affective impact of such an extensive set of words introduced in a situational context has been found in the literature. The mid-term objective is to deliver a formal corpus that can provide a ground definition for suspenseful terms, as part of the development of a computational model of suspense to predict the effect of texts in the audience (Delatorre et al., 2016a). In this line, the long-term aim is to improve the model to predict the emotional effect of complex concepts when they are introduced in different contexts, not restricted to suspense. Additionally, we reckon this may be the precursor of similar future works, to contrast Redondo et al. (2007) ANEW with different contexts, to expand the set of words, or to analyze the effects of suspense in different narrative media.

## Data Availability

The datasets generated for this study are available on request to the corresponding author.

## Ethics Statement

This study was carried out in accordance with the recommendations of national and international ethics guidelines, Código Deontológico del Psicólogo and American Psychological Association. This study did not present any invasive procedure, and it did not carry any risk to the participants' mental or physical health, thus not requiring ethics approval according to the Spanish law BOE 14/2007. All subjects participated voluntarily and gave written informed consent in accordance with the Declaration of Helsinki. They were free to leave the experiment at any time.

## Author Contributions

PD, AS, CL, and AT contributed to the conception of the study, designed the experimental method, and wrote and approved the version to be published. PD investigated and sorted the related work and interpreted the findings. PD and AS conducted the experiment and collected the data.

### Conflict of Interest Statement

The authors declare that the research was conducted in the absence of any commercial or financial relationships that could be construed as a potential conflict of interest.

## Footnotes

^1^This is currently one of the main difficulties in providing a computational model and an implementation of suspense in automatic storytelling. Due that no complete formal specification of the constituents and impact of suspense has still been proposed, it is a challenge to develop formal processes. Actually, the automatic story generation systems including suspense evaluate and implement it with a limited functional simplification, for example implementing some emotional links between the characters or reducing the chances of success for the protagonists in certain situations (Turner, 2014; Pérez y Pérez, 1999; Cheong and Young, 2006; Szilas, 2007; O'Neill and Riedl, 2014). Ultimately, the lack of a formalism results in the fact that several potential features of the different conceptualizations of the term are still not explicitly addressed, such as the quantitative measures of emotions during the referred process of anticipation of the outcome.

^2^“Context of suspense” is defined as meaningful scene boundaries that promote the emergence of suspense.

^3^University of Cadiz, Complutense University of Madrid, and University of Jaén.

^4^*dif* standing for “difference of means.”

^5^Respectively *v* and *a* standing for *valence* and *arousal*.

^6^Due to word relations and in order to simplify, we decided to included *pleasure* as relevant to sexual activities, although it could be considered as relative to general well-being. This criterion may be applied to other terms from the lists, due to the inherent difficulty associated to disambiguate the intended connotations of the subjects. In any case, we consider this does not represent a significant difference in the general analysis.

^7^As Ullrich et al. (2017) exposes, it remains an open question whether one single word with an extreme affective value could even dominate the affective perception of the whole background.

## Supplementary Material

The instructions provided to the experimental subjects, and the contextualized Spanish S-ANEW corpus are included as Supplementary Material.
