# Supplementary material for: The Impact of Context on Affective Norms: A Case of Study With Suspense
Source: Front Psychol. 2019 Aug 30;10:1988. doi: 10.3389/fpsyg.2019.01988 (PMC6728922; doi:10.3389/fpsyg.2019.01988)
Supplement: Supplementary file 2 [file Data_Sheet_2.PDF]

[TRANSLATION OF THE ORIGINAL INSTRUCTIONS, IN SPANISH]

Please, read this whole text sequentially before moving on to the next pages, even if they are specifically mentioned early on. The wrong interpretation of the text or simply reading ahead can produce errors in the results.

This study concerns emotions and suspense. Specifically, we are trying to find out which emotions are evoked by words when these words are introduced in a scene of suspense.

Thus, your task is to evaluate how you feel when you read each of the following words, considering that each term appears in a suspenseful scene. In this sense, picture the ”suspenseful scene” as a situation in which a victim is nearing an event with a harmful outcome due to a threat, as commonly found in suspense films and novels. The words to be rated are independent to each other. Likewise, the imagined scene may be very simple, and it can optionally be based on some other scene you previously watched or read. Also, the scene does not need to be the same for all the words.

In order to assess your feelings when you read the words, we are going to use a character, SAM, who can represent three types of feeling: pleasant/unpleasant emotions (the first series, on the left, called *valence*), excitation/calm (the second series, on the middle, called *arousal*), and in-control/out-of-control feeling (the third series, on the right, called *dominance)*.

For the valence scale, we ask you to estimate how much the given word makes you feel something positive or negative. If you feel completely pleased, contented, or hopeful, you should mark the value 9 in the scale (very pleasant). However, if you feel completely displeased, unsatisfied, or hopeless, you should mark the value 1 in the scale (very unpleasant). If you feel completely neutral, neither pleased nor displeased, you should mark the value 5 in the scale.

Regarding the arousal scale, we ask you to estimate how much the given word makes you feel something arousing or calming, whether the word makes you feel pleased or displeased. If you feel completely stimulated, excited, frenzied, jittery, wide-awake, or aroused, you should mark the value 9 in the scale (very arousing). However, if you feel completely relaxed, calm, sluggish, dull, sleepy, or unaroused, you should mark the value 1 in the scale (very calming). If you feel completely neutral, neither excited nor calmed, you should mark the value 5 in the scale.

Finally, for the dominance scale, we ask you to estimate to what extent the given word makes you feel something dominant or submissive. If you feel completely in control, influential, important, dominant, autonomous, or controlling, you should mark the value 9 in the scale (very dominant). However, if you feel completely controlled, influenced, cared-for, awed, submissive, or guided you should mark the value 1 in the scale (very submissive). If your feel completely neutral, neither in control nor controlled, you should mark the value 5 in the scale.

Summarizing, you should rank whether a specific word makes you feel pleased/displeased, excited/calmed, and dominant/submissive, when the word is introduced in a suspenseful scene, by marking with an X in the corresponding column scale, only one for each word-dimension pairing, as shown in the examples sheet.

Please, fill in the questionnaire in a calmed location with as little distractions as possible, using as many sessions as required. It is important to answer as spontaneously as possible, using the

desired ratings from the whole range. Try not to think too much; base your answer on your first reaction to the word. There are no right or wrong answers. The focus should be on how you feel when you read the word as it was introduced in a suspenseful scene.

We thank your collaboration.
